# Live Biotherapeutic Products for Metabolic Diseases: Development Strategies, Challenges, and Future Directions

**DOI:** 10.4014/jmb.2410.10054

**Published:** 2025-03-11

**Authors:** Heonhae Min, Kyu-Sung Choi, Saebom Yun, Sungho Jang

**Affiliations:** 1 Department of Bioengineering and Nano-Bioengineering, Incheon National University, Incheon 22012, Republic of Korea; 2 Division of Bioengineering, College of Life Sciences and Bioengineering, Incheon National University, Incheon 22012, Republic of Korea; 3 Research Center for Bio Materials and Process Development, Incheon National University, Incheon 22012, Republic of Korea

**Keywords:** Metabolic diseases, gut microbiome, live biotherapeutic products

## Abstract

Metabolic diseases, such as obesity, type 2 diabetes, and non-alcoholic fatty liver disease, have emerged as major global health challenges. Recent research has revealed that the gut microbiome is closely associated with the development of these conditions. The Food and Drug Administration has recognized certain probiotic strains with therapeutic potential, classifying them as live biotherapeutic products (LBPs). LBPs, which are derived from naturally occurring microorganisms, may present an effective strategy for treating metabolic diseases by restoring gut microbiota balance and regulating metabolic functions. This review explores the development of LBPs specifically for metabolic disease treatments, covering every phase from strain identification, non-clinical and clinical trials, manufacturing and formulation to regulatory approval. Furthermore, it addresses the challenges involved in the commercialization of these therapies. By offering critical insights into the research and development of LBPs for metabolic disease treatment, this review aims to contribute to the progress of these promising therapies.

## Introduction

The gut microbiome, consisting of a vast and diverse community of microorganisms within the gastrointestinal tract, plays a crucial role in maintaining human health. These trillions of microorganisms function not merely as “inhabitants” but as key contributors to both physical and mental well-being. The gut microbiome supports human health by aiding in food digestion, facilitating nutrient absorption, maintaining normal immune function, enhancing defense mechanisms against pathogens, synthesizing essential vitamins like B and K, and detoxifying harmful substances [1-3]. Additionally, through the microbiota-gut-brain axis, it has a significant impact on brain activity and cognitive functions [4].

Live biotherapeutic products (LBPs) have recently garnered considerable attention for their potential in treating diseases linked to the gut microbiome. The Food and Drug Administration (FDA) defines LBPs as “a biological product that: 1) contains live organisms, such as bacteria; 2) is applicable to the prevention, treatment, or cure of a disease or condition of human beings; and 3) is not a vaccine” [5]. LBPs and probiotics share similarities in their definitions and applications, which can lead to confusion. Probiotics are defined as “live microorganisms that, when administered in adequate amounts, confer a health benefit on the host.” While both LBPs and probiotics aim to promote health, LBPs are classified as medicinal products that specifically target certain diseases and must demonstrate both safety and efficacy.

In contrast, probiotics are typically classified as dietary supplements and are subject to less stringent regulations. If proven effective in treating diseases, probiotics may be reclassified as LBPs. Furthermore, LBPs include recombinant LBPs as a subcategory. Recombinant LBPs consist of genetically modified microorganisms that have undergone the intentional addition, deletion, or modification of genetic material. Non-recombinant LBPs, on the other hand, are generally referred to simply as LBPs without further distinction. Unlike recombinant LBPs, non-recombinant LBPs utilize naturally occurring microorganisms selected for their genetic stability and may face fewer regulatory hurdles during development and commercialization [6]. LBPs hold significant potential, particularly in treating metabolic diseases. Conditions, such as obesity, type 2 diabetes mellitus (T2DM), and non-alcoholic fatty liver disease (NAFLD), are closely linked to imbalances in the gut microbiome. LBPs offer innovative preventive and therapeutic solutions by restoring microbial balance in the gut, reducing inflammation, enhancing immune function, and optimizing metabolism processes. This approach has added benefit of fewer side effects compared to conventional therapies, promoting the restoration of the natural balance of body. Certain probiotic strains have shown potential in reducing body weight and improving insulin sensitivity in obese patients, enhancing glycemic control in diabetic patients, and providing therapeutic benefits for those with NAFLD [7-10].

**Fig. 1:**
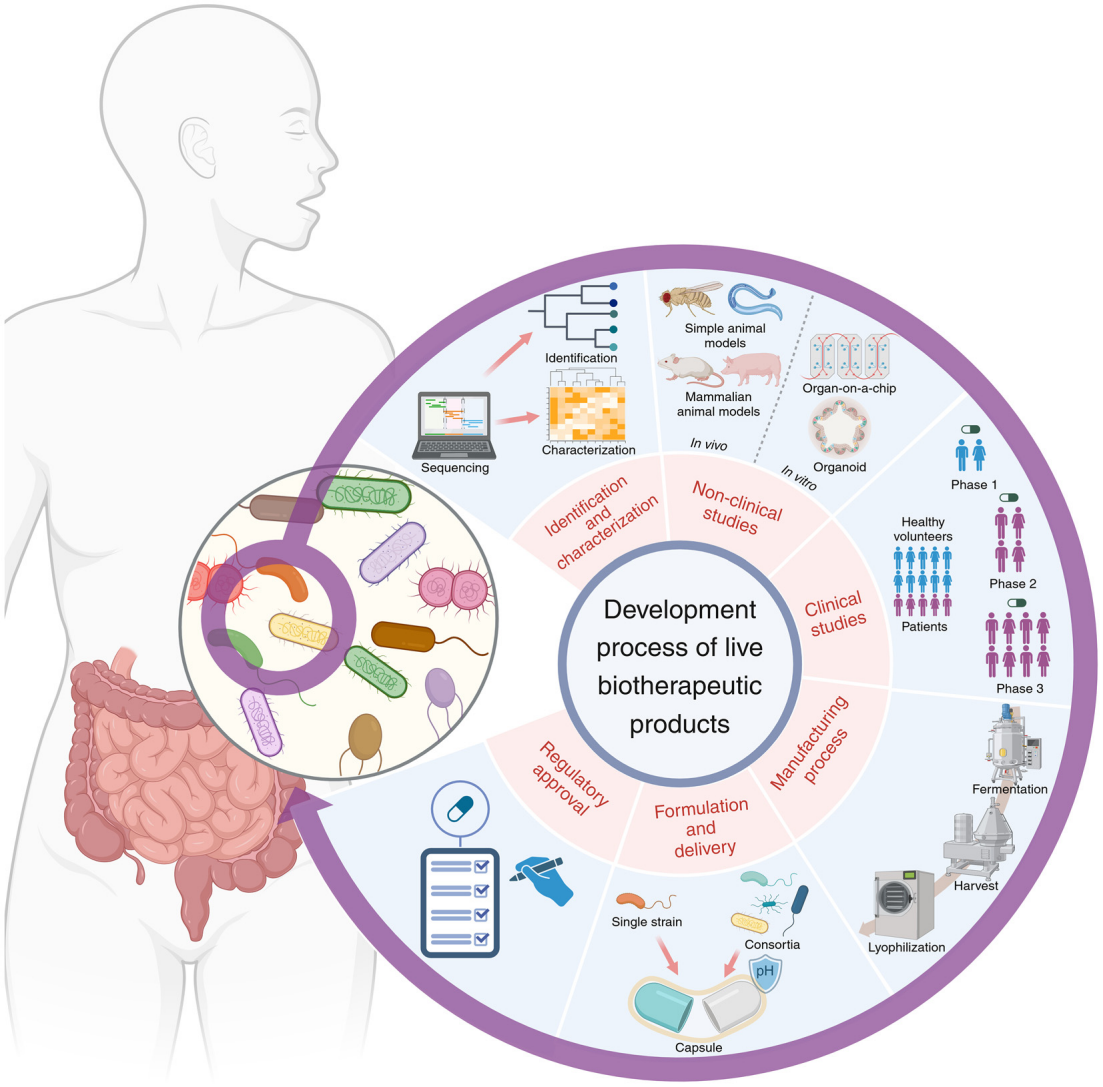
Schematic overview of the development process of LBP. The development of LBPs progresses through several critical phases, initiating from strain identification and characterization, then advancing through non-clinical studies and clinical trials. Subsequently, the product undergoes manufacturing optimization and formulation development, and appropriate delivery systems are established. Regulatory approval processes evaluate all aspects of these development stages, ultimately yielding a market-ready therapeutic.

This review focuses on the different stages of LBP development, excluding recombinant LBPs, with a particular emphasis on the identification and functional characterization of naturally occurring microbial species or communities that are effective in treating diseases. It provides a detailed overview of the entire LBP development process, covering non-clinical and clinical studies, regulatory approval procedures, manufacturing processes, as well as formulation and delivery methods (Fig. 1).

## LBPs for Metabolic Diseases

### Metabolic Diseases associated with the Gut Microbiome

Metabolic diseases are closely linked to the human gut microbiome, with the composition and alterations of gut microbial communities playing a critical role in the risk of developing these conditions [11]. This connection between the gut microbiome and metabolic diseases highlights the potential therapeutic applications of LBPs. In this section, key metabolic diseases associated with the gut microbiome are discussed.

Obesity, a prevalent metabolic disease worldwide, has been increasingly associated with the gut microbiome. Recent studies suggest that gut microbes significantly contribute to obesity by fermenting dietary fibers into short-chain fatty acids (SCFAs), which enhance energy harvest and promote fat accumulation [12, 13]. Research involving germ-free mice inoculated with microbiota from obese individuals showed a notable increase in body fat compared to mice inoculated with lean microbiota [14]. Furthermore, individuals with a higher body mass index (BMI) exhibited a significantly lower ratio of secondary to primary bile acids, suggesting that the gut microbiome may regulate bile acid metabolism and influence metabolic processes linked to obesity [15].

An imbalance in the gut microbiome can contribute to the development of T2DM. An increase in harmful microbes within the gut microbiome promotes intestinal inflammation and weakens the gut barrier, allowing inflammatory substances to enter the bloodstream. This triggers systemic inflammation, which disrupts insulin signaling pathways and heightens insulin resistance. Prolonged insulin resistance can impair blood glucose regulation, potentially leading to the onset of T2DM [16, 17].

NAFLD encompasses a spectrum of conditions, ranging from non-alcoholic fatty liver (NAFL), which is marked by the absence of inflammation, to non-alcoholic steatohepatitis (NASH), which involves inflammation and liver cell injury. Over time, NAFLD can progress to fibrosis, cirrhosis, and hepatocellular carcinoma [18, 19]. The gut microbiota and liver are closely connected through a bidirectional relationship known as the gut-liver axis [20]. In patients with NAFLD, gut microbial diversity is significantly reduced, while specific microbial groups, such as *Desulfovibrio*, *Negativibacillus*, and *Prevotella*, are increased [21]. A study demonstrated that transplanting lipopolysaccharide (LPS)-producing strains, including *Enterobacter cloacae* B29, *Escherichia coli* PY102, and *Klebsiella pneumoniae* A7, isolated from the gut of patients with hepatic steatosis, into germ-free mice accelerated the development of NAFLD [22]. These gut microbes can promote inflammation and fat accumulation in the liver, underscoring the importance of modulating the gut microbiota as a therapeutic target for treating NAFLD [10].

### The Potential of Probiotics with Established Mechanisms of Action (MoA) in the Development of LBPs for Metabolic Disease Treatment

As the role of the gut microbiome in treating metabolic diseases becomes increasingly clear, the demand for developing LBPs based on this knowledge is growing. LBPs, whether composed of single or multiple microbial species, can be used to address metabolic diseases, and their development has been actively pursued in recent years [23, 24]. However, the application of LBPs in treating metabolic diseases remains limited, with few examples of commercialized products. This is largely due to several challenges, including the complexities of regulatory and safety validations in LBP development, as well as uncertainties about interactions between different microorganisms.

In contrast, the therapeutic mechanisms of certain probiotics for metabolic diseases have already been identified, indicating a strong potential for developing effective LBPs using these probiotics. The following are examples of probiotics with known therapeutic mechanisms (Table 1). Anti-obesity and weight management begin by reducing food intake. *Hafnia alvei* HA4597 helps regulate weight by increased the secretion of the peptide YY (PYY) hormone in the gut, which suppresses appetite and induces a feeling of satiety [25-27]. Furthermore, *Lactobacillus rhamnosus* JL1 reduced body weight by activating the AMP-activated protein kinase (AMPK) pathway and decreasing the gene expression of peroxisome proliferator–activated receptor-γ (PPAR-γ), liver X receptor α (LXR-α), and sterol regulatory element binding protein 1c (SREBP-1C) [28]. Additionally, *Akkermansia muciniphila* produces P9, a compound that promotes the secretion of glucagon-like peptide-1 (GLP-1), enhances thermogenesis and glucose homeostasis, and ultimately leads to weight reduction [29].

As a strain that may assist in treating T2DM, *A. muciniphila* not only exhibits anti-obesity properties but also stimulates the secretion of hormones such as incretin, which enhances insulin sensitivity. It contributes to improving intestinal wall integrity, thus preventing the translocation of inflammatory substances. Additionally, it helps regulate glucose tolerance and lipogenesis, which in turn improves blood glucose and lipid metabolism [30-32]. *L. plantarum* CCFM0236 improves insulin resistance by inhibiting α-glucosidase activity, regulating antioxidant capacity, and modulating inflammatory responses, leading to reduced blood glucose levels [33]. *L. casei* enhances the expression of serum sirtuin1 (SIRT1) while decreasing fetuin-A levels, thereby improving insulin resistance and glycemic control, which results in weight reduction for patients with T2DM [34].

NAFLD is closely linked to dysbiosis of the gut microbiota, characterized by an increase in harmful bacteria that contribute to insulin resistance and alterations in lipid metabolism. Factors such as obesity and insulin resistance are commonly associated with the onset of NAFLD [35]. *Bifidobacterium lactis* V9 activates the AMPK pathway to promote fatty acid oxidation and inhibits the toll-like receptor-nuclear factor κB (TLR-NF-κB) pathway, effectively reducing hepatic lipid accumulation and inflammation [36]. Meanwhile, *L. plantarum* NCU116 enhances the expression of fatty acid oxidation genes and suppresses the expression of lipogenesis genes in the liver, thereby decreasing fat accumulation and improving the condition of NAFLD [37].

## Development Procedure for LBPs

LBPs have significant potential for treating diseases through the use of microorganisms, but their development process presents unique challenges that differ from those faced by traditional drugs and biopharmaceuticals. LBPs are often developed using a mixture of various microbial compositions, which complicates the achievement of the level of microbial community characterization required for regulatory approval. The dynamic nature of these living organisms adds complexity that is not typically observed with conventional drugs. Unlike traditional medications, the exact mechanisms by which LBPs exert their therapeutic effects in the human body may remain unclear. Furthermore, given that gut microbiota composition varies among individuals, an LBP that is effective for most people might not perform as well in certain patients, potentially leading to adverse effects [38]. To ensure the therapeutic efficacy and *in vivo* stability of LBPs, special handling may be necessary for their distribution and storage conditions, posing challenges in regions with limited infrastructure that cannot meet these specific requirements. Developing LBPs necessitates new approaches and presents various challenges. This section outlines methods for developing LBPs, emphasizing the importance of establishing causality rather than merely correlation between diseases and the microbiome. We present guidelines for LBP development, which include the identification and functional characterization of microbial species or communities with therapeutic potential from the natural microbiome, as well as multi-omics approaches and non-clinical studies, as key steps in effective LBP development.

### Identification and Characterization

Identification and characterization are critical initial steps in the development of LBPs. This process is vital for evaluating the therapeutic efficacy of LBPs in treating diseases and ensuring their safety to prevent adverse effects in patients. The emphasis is on screening and selecting potential microorganisms from natural sources that can be candidates for use in LBPs.

**16S rRNA sequencing.** The 16S rRNA has been widely utilized for the identification of prokaryotes and serves as a molecular phylogenetic marker capable of distinguishing different bacterial species. All prokaryotes, including bacteria, possess the 16S rRNA gene, which has a sequence length of approximately 1,500 bp. This sequence is sufficiently conserved to identify a broad range of microorganisms while remaining variable enough among different species to allow for differentiation. Techniques such as 454 pyrosequencing and the Illumina MiSeq platform analyze the hypervariable regions of this gene, facilitating taxonomic identification and the detection of unknown sequences [39]. 16S rRNA sequencing is increasingly employed to analyze microbial communities associated with metabolic diseases, particularly focusing on obesity and diabetes. Research in obesity encompasses the examination of microbial compositions in the duodenum associated with obesity [40], the classification of microbes through 16S rRNA profiling [41], and studies highlighting differences in gut microbiota between obese and normal-weight individuals [42]. Research related to diabetes includes comparisons of gut microbiota in elderly individuals with glucose metabolism diseases using 454 sequencing [43], analyses of gut microbiota changes following 3-bromo-4,5-bis(2,3-dibromo-4,5-dihydroxybenzyl)-1,2-benzenediol (BDB) administration in a T2DM mouse model [44], and investigations into the effects of probiotic administration on gut microbiota in a gestational diabetes rat model [45].

**Metagenome sequencing.** Metagenome sequencing, which analyzes the genetic composition of entire microbial communities, can be utilized to select microorganisms for LBPs. This method provides the advantage of obtaining comprehensive genetic information without the need to culture and analyze each microorganism individually, as it allows for the extraction and sequencing of all microbial DNA present in a specific environment or sample. Furthermore, metagenome sequencing serves as an efficient tool for understanding the genetic composition and functional capabilities of complex microbial communities, aiding in the discovery of previously unknown microorganisms. For instance, a study revealed structural changes in gut microbiota associated with obesity, highlighting the differences in gut microbiota composition among obese patients that could be utilized in the development of personalized LBPs [46]. Similarly, analyzing gut microbiota and metabolites enables early identification of stroke risk [47] and, when combined with polygenic risk scores (PRSs), it facilitates the prediction of risks for common diseases such as T2DM and coronary artery disease (CAD) [48]. Metagenome sequencing can be integrated with other research methods to enhance applications in disease treatment.

**MALDI-TOF MS.** Matrix-assisted laser desorption/ionization-time of flight mass spectrometry (MALDI-TOF MS) is a mass spectrometry technique used to ionize proteins or other molecules in various samples and measure their mass for analysis. Unlike sequencing-based techniques, which require preprocessing, this method allows for rapid identification of microbial strains by comparing the unique protein spectrum of a specific microorganism to a database. Additionally, MALDI-TOF MS is cost-effective and capable of analyzing a wide range of microorganisms, including bacteria, fungi, and viruses [49]. However, the technique has certain limitations. If the database is insufficient, it may struggle to accurately differentiate species due to incomplete or inadequate reference spectra. Moreover, it may have difficulty differentiating between closely related species, such as *E. coli* and *Shigella* [50]. Despite these challenges, MALDI-TOF MS has been applied in various fields. For instance, it has been empirically validated for the rapid and accurate identification of microorganisms in clinical samples, such as *Streptococcus salivarius* and *Lactobacillus* species [51]. Furthermore, the technique has been employed to identify microbial strains in dietary supplements, ensuring product quality and labeling accuracy [52].

The ability of MALDI-TOF MS to identify various microbial communities during the manufacturing process of LBPs plays a critical role in quality control of LBP production [53]. Furthermore, the integration of MALDI-TOF MS with a mass spectral profile (MSP) library has demonstrated the rapid and accurate identification of human gut symbiotic bacteria. This integrated approach illustrates how MALDI-TOF MS can be expanded and enhanced when combined with other research methodologies [54].

**Multi-omics.** Multi-omics is an approach that extends beyond simple genome analysis by integrating various biological data (such as transcriptomics, proteomics, metabolomics) to achieve a comprehensive understanding of biological phenomena. For example, transcriptomic analysis reveals gene expression patterns of specific microorganisms, proteomics assesses protein expression, and metabolomics identifies microbial metabolites. Recent studies have demonstrated that analyzing the multidimensional characteristics of microbial communities related to obesity and metabolic diseases provides crucial information for designing personalized LBPs [55]. Additionally, the multi-omics approach can be utilized to decipher complex interactions within gut microbial communities and uncover new therapeutic targets. For instance, a study used 16S rRNA sequencing and metagenomic analysis to identify the composition of gut microbiota, metatranscriptomics to analyze gene expression, and lipidomics to evaluate fecal metabolites, elucidating the relationship between gut microbiota and dietary habits in obese women [56]. Such integrated analyses can reveal how specific metabolites are linked to diseases and assist in selecting microorganisms with therapeutic potential [56, 57].

### Non-Clinical Studies

As discussed above, after classifying and analyzing the functions of the microbiome using molecular techniques and data analysis, it is essential to validate these findings experimentally before clinical application. Given that LBPs involve complex direct or indirect interactions within humans [58], understanding their mechanism of action from multiple perspectives through non-clinical studies is crucial. Various methods and techniques have been developed to effectively evaluate these interactions in both *in vivo* and *in vitro* settings.

***In vivo* studies.**

***Simple animal models.*** One approach to studying how LBPs interact within the host gut *in vivo* is the use of simple animal models. Models such as *Drosophila melanogaster* (vinegar fly) and *Caenorhabditis elegans* (nematode worm) offer several advantages over mammalian models, including reduced experimental time, lower costs, and the ability to design more complex experiments with greater ease [59]. While the gut structure and function of *Drosophila* differ from those of humans, conserved metabolic pathways exist [60], and its relatively simple gut microbiota, consisting of 1–30 species [61], makes it a suitable model for studying microbiome-host interactions. *C. elegans* provides the added benefit of being easy to genetically manipulate, making it well-suited as a gnotobiotic model [62]. Moreover, *C. elegans* is ideal for studying host-microbe interactions, as its gut microbiome can be used to study the effects on diet, lipid metabolism, the nervous system, drug metabolism, and even aging [63]. Although invertebrate experiments may be challenging to directly translate to human biology, they raise fewer animal welfare concerns and can be effectively utilized in LBP experiments or screenings.

***Mammalian animal models.*** Mammalian animal models are well-suited for predicting the *in vivo* responses of LBPs in humans because of their physiological and immunological similarities to the human system. Among these models, mice are most commonly used for testing LBPs [64]. Mice have served as the primary model in studies exploring the interactions between gut microbiota and the host, leading to the development of various mouse models. These include specific pathogen-free (SPF) mice, antibiotic-treated mice, germ-free (GF) mice, and gnotobiotic mice, all of which have been employed in gut microbiota research. Of these, the gnotobiotic mouse model is particularly effective and widely used as a platform for microbiome studies. Beyond mice, other mammalian models are also utilized in LBP research [65]. For instance, pigs offer valuable insights into the relationship between gut microbiota and metabolic diseases due to their physiological similarities with the human gastrointestinal tract [66].

***In vitro* studies.** *In vitro* models are essential tools for analyzing the mechanism of action of LBPs at the tissue level within a human physiological context. Systems like organoids and organ-on-a-chip can simulate specific disease conditions by closely mimicking the human environment. These models help address the limitations of animal models, such as physiological differences from humans and ethical concerns, making them valuable for LBP development.

***Organoids.*** Since the development of self-organizing, three-dimensional intestinal organoids derived from leucine-rich repeat-containing G protein-coupled receptor 5+ (LGR5+) intestinal stem cells in 2009 [67], organoids have emerged as a promising tool for modeling various human organs. Organoids can be generated from different cell types, including embryonic stem cells (ESCs), induced pluripotent stem cells (iPSCs), adult stem cells, and cancer cells. Their three-dimensional structure consists of epithelial tissue surrounding a lumen, which contains mucus and cells. Because bacteria reside within the lumen, the 3D architecture has made it challenging to access the interior for microbiome research [68]. However, the development of microinjection techniques, which enables the delivery of microbes and other cargo into the lumen of organoids, has advanced microbiome studies in these models. Despite this progress, this method remains technically demanding and has limitations in accuracy [69].

***Organ-on-a-chip.*** Organ-on-a-chip is a microfluidic-based device designed to replicate the cells, tissues, and functions of *in vivo* organs, thereby recreating physiological processes. These platforms integrate various technologies, including lab-on-a-chip systems, microfluidic devices, and cell biology, making them increasingly promising models for *ex vivo* disease research, drug response testing, and toxicity and efficacy evaluation [70]. A wide range of organ-mimicking devices has been developed using organ-on-a-chip technology, such as lung-on-a-chip [71], heart-on-a-chip [72], liver-on-a-chip [73], brain-on-a-chip [74], kidney-on-a-chip [75], blood vessel-on-a-chip [76], skin-on-a-chip [77], tumor-on-a-chip [78], bone marrow-on-a-chip [79], and gut-on-a-chip [80], among others. In one study, a high-throughput gut-on-a-chip model was used to mimic key aspects of inflammatory bowel disease [81]. Based on Caco-2 cells, the platform tested the effects of cytokine triggers on intestinal barrier integrity and intestinal epithelial cell (IEC) activation. Treatment with the anti-inflammatory compound 2-[(aminocarbonyl)amino]-5-(4-fluorophenyl)-3-thiophenecarboxamide (TPCA-1) significantly suppressed cytokine production, reducing inflammation. Gut-on-a-chip is particularly well-suited for evaluating how LBPs interact within the human gut, as it specializes in replicating the gut and its microbiome. This technology continues to be refined to better mimic the complex structure and functions of the gut, addressing current limitations [82].

### Clinical Studies

When non-clinical studies suggest that LBPs have the potential for promising therapeutic outcomes, clinical studies in humans are necessary to confirm their safety and efficacy. Clinical research is typically divided into three phases. The first safety assessment (Phase I) involves the safety and tolerability of LBPs in healthy volunteers, with a particular focus on managing the risk of systemic infection due to the live bacterial nature of LBPs. Currently, several clinical trials are underway to evaluate the safety and efficacy of LBPs for treating metabolic diseases. For example, YSOPIA Bioscience’s single-strain LBP, Xla1, is being tested in an open-label study with healthy volunteers and a placebo-controlled trial in overweight or obese adults, monitoring safety, tolerability, and its impact on the microbiome [23]. Similarly, NuBiyota’s multi-strain LBP, MET-3, is being evaluated in a randomized, uncontrolled pilot study with obese adults to explore the metabolic effect. This study focuses on assessing its safety, efficacy of MET-3 in obese adults [24]. The early efficacy assessment (Phase II) evaluates the therapeutic effects and determines the optimal dose in the target patient population, aiming to establish a causal relationship between product administration and disease symptom improvement. The European Medicines Agency (EMA) favors demonstrating the mechanism of action of the product, as it aids in patient monitoring and helps understand the drug’s effects before entering later trial stages. The final confirmation and comparison phase (Phase III) involves larger scale testing to confirm the safety and efficacy of LBPs across diverse populations, comparing their benefits with existing treatments to establish them as a viable therapeutic option [83].

### Manufacturing Process

The manufacturing process of LBPs necessitates careful management of microbial complexity and variability. It begins with the selection of a strain with demonstrated efficacy, followed by genomic analysis and functional assays to assess its characteristics. After optimizing culture conditions to ensure a high-quality strain, large-scale production is conducted through fermentation, with the strain harvested via centrifugation and filtration. Post-processing steps, including purification, concentration, and lyophilization, are implemented to remove impurities, enhance stability, and ensure consistency and safety through quality control and assurance procedures such as antibiotic resistance testing and impurity analysis [84, 85]. A comprehensive risk assessment is performed to manage strain mobility and pathogenicity, thereby ensuring the safety of the final product [86]. For anaerobic strains, more specialized culture and management conditions are required. This involves selecting strains that thrive in anaerobic environments and establishing culture systems that can maintain these conditions. Oxygen levels are rigorously controlled throughout this process to optimize strain growth. Once the anaerobic culture process is complete, the strain is harvested through centrifugation and filtration, with anaerobic conditions preserved during subsequent purification and concentration steps. To enhance the stability of anaerobic strains, specialized packaging and storage conditions must be implemented, and quality control procedures must reflect these requirements [87-89].

### Formulation and Delivery

Developing the appropriate formulation and delivery method is a crucial final step for the effective therapeutic application of LBPs. During this development, conditions are optimized to maximize microbial viability, stability, and activity. Oral administration is the most common approach, necessitating formulations that protect the microbes from stomach acid and digestive enzymes, often using enteric coatings for targeted release in the intestines [90]. Recently, innovative encapsulation technologies, such as sensitive delivery systems, multilayer encapsulation, and polyelectrolyte complexation, have been developed to enhance the biocompatibility, biodegradability, and stability of LBPs [91]. These methods demonstrate promising results in maintaining high viability and ensuring the targeted release of active microbes, thereby improving delivery efficiency and therapeutic outcomes in the treatment of metabolic diseases.

### Regulatory Approval

For a successful market release, LBPs must receive approval from regulatory authorities such as the U.S. FDA or the European EMA. All clinical trial results, along with data on manufacturing process consistency, cleanliness, and product quality control systems, must be submitted. Due to their unique biological nature, LBPs must comply with stringent good manufacturing practice (GMP) conditions during manufacturing to ensure microbial viability and prevent contamination, while also providing high-quality clinical trial data demonstrating efficacy and safety. Particularly for LBPs, since live microorganisms are the main component, the impact of formulation and delivery methods on the stability of the microorganisms must be thoroughly evaluated. Regulatory authorities will review the submitted data and may request additional information. Once all requirements are satisfied, LBPs can be approved for market release [92]. Approved LBPs, backed by clinically proven therapeutic efficacy and safety, can offer patients a new treatment option.

## Conclusion and Future Directions

Numerous studies involving gut microbiota have led to the commercialization of products that enhance human health. LBPs have gained attention as therapeutic agents capable of treating diseases, moving beyond the traditional concept of probiotics. As a result, efforts have been made to apply LBPs to various conditions, including gastrointestinal diseases and metabolic diseases. Recently, two microbiome-based therapeutics have been approved, both aimed at preventing recurrent *Clostridioides difficile* infection, a serious gastrointestinal condition often exacerbated by antibiotic use. Rebyota (formerly known as RBX2660), developed by Rebiotix, was approved by FDA as the first microbiome-based therapy in 2022 [93]. It is comprised of a broad mixture of microorganisms derived from human donor stool, functioning similarly to fecal microbiota transplantation (FMT) in that it aims to restore gut microbiota. On the other hand, Vowst (previously known as SER-109), developed by Seres Therapeutics, is an oral capsule containing purified spores of selected bacterial species derived from the fecal matter of healthy donors, making it a more defined and targeted approach than Rebyota [94]. Unlike Rebyota and Vowst, LBPs are typically composed of carefully selected microbial strains or consortia designed for specific therapeutic functions and cultured for large-scale production, rather than relying on a broad mixture of donor-derived microbes. LBPs for the treatment of metabolic diseases focuses on introducing specific beneficial microorganisms that can produce therapeutic effects through well-elucidated mechanism of action. It aims to reduce inflammation [95], produce beneficial metabolic byproducts, and enhance immune function to address diseases [96]. Additionally, LBPs can directly combat diseases by producing specific proteins [29]. However, due to the complex interactions of living microbes and individual variability in gut microbiota composition, predicting and standardizing the therapeutic effects of LBPs remains challenging. Therefore, for LBPs aimed at treating metabolic diseases, understanding the diversity of the gut microbiome and its interactions is crucial. Consequently, meeting the stringent and complex requirements set by regulatory agencies to gain approval as a therapeutic may pose challenges, creating significant barriers to advancing beyond the clinical trial development stage.

The development of LBPs involves multiple stages, including microbial identification and characterization, non-clinical and clinical studies, regulatory approval, manufacturing process development, and optimization of formulation and delivery methods. The identification and characterization of microbes is a crucial step in this development process. Specific strains with therapeutic potential for certain diseases are selected from naturally derived microbes, and their functional characteristics are analyzed using techniques such as 16S rRNA sequencing, metagenomic sequencing, and multi-omics approaches. The selection of strains with established therapeutic effects is essential for the success of the development process. During the non-clinical and clinical study phases, various experiments are conducted to demonstrate the safety and efficacy of LBPs. The mechanism of action of the microbes are investigated using *in vivo* animal models and *in vitro* organoid or organ-on-a-chip models, while clinical trials confirm the safety and effectiveness of LBPs in humans. In the development stage of the manufacturing process, optimal culture conditions are established to maintain microbial viability and activity, while the product’s quality is ensured through purification and lyophilization processes. Anaerobic strains, in particular, require special handling. The development stage of formulation and delivery method focuses on creating appropriate formulations and delivery systems to maximize the therapeutic effect of LBPs. Techniques such as enteric coating for oral administration and advanced encapsulation technologies are employed to enhance microbial survival and maintain activity at the target site. Among these stages, the selection of strains through microbial identification and characterization is the most crucial. While discovering new strains is an important strategy, selecting probiotic strains with proven efficacy for the target disease is a more efficient approach that can save time and costs. Regulatory approval is the mandatory step for the commercialization of LBPs. At each stage of development and production, regulatory authorities rigorously evaluate the consistency, safety, and stability of the manufacturing process. Approval for commercialization is granted based on the results of clinical studies, ensuring that LBPs meet the necessary standards for patient safety and product efficacy. Therefore, consideration of regulation is crucial at every stage of the LBP development procedure to ensure successful progression toward commercialization.

The future development of LBPs should proceed in several key directions. First, given the variability in gut microbiota among individuals, there is a pressing need for personalized LBPs that take individual characteristics into account. By analyzing an individual’s gut microbiota through microbiome profiling and designing optimized LBPs, it may be possible to treat diseases like metabolic diseases more effectively, as these conditions can present differently in each person [97]. Second, there is a necessity for developing LBPs based on complex microbial consortia. Currently, LBP development is primarily focused on single-strain products. LBPs derived from complex microbial consortia could potentially provide greater benefits than single-strain formulations due to microbial interactions and resulting synergistic effects. For example, such consortia could enhance microbial viability by facilitating the exchange of different metabolic products, inducing optimal pH ranges, or creating anaerobic environments [98]. Additionally, a study demonstrated that the combination of *L. plantarum* KY1032 and *L. curvatus* HY7601 was more effective than single strains in preventing fat accumulation in adipose tissue and the liver [99]. This suggests that multi-strain probiotics may be more effective in mitigating obesity-related metabolic changes. Third, the long-term effects of LBPs on the gut microbiome should be investigated. Research on the long-term efficacy and safety of LBPs is currently limited. Future studies should prioritize tracking changes in the gut microbiome following LBP administration to ensure sustainable effects and long-term safety.

In conclusion, although LBPs may pose challenges during development, they offer a novel approach to treating metabolic diseases that distinguish them from traditional pharmaceuticals, serving as a valuable alternative. With ongoing research and development, LBPs have the potential to evolve into more refined and safer therapeutic options, ultimately aiding many patients in regaining their health and improving their quality of life.

## Figures and Tables

**Table 1:** Current Status of LBPs in the Treatment of Metabolic Diseases.

Organism	Disease	Model	Result	Reference
*H. alvei* HA4597	Obesity	ob/ob mice	Decreased body weight, ameliorated plasma glucose and total cholesterol levels	[25]
	Obesity	ob/ob mice	Decreased body weight, reduced fat mass, lowered food intake in hyperphagic mice	[26]
	Obesity	236 overweight	adults Improved weight loss, increased fullness, reduced hip circumference	[27]
*L. rhamnosus* JL1	Obesity	High-fat diet mice	Decreased body weight, improved liver lipid metabolism, reduced serum total cholesterol (TC), triglycerides (TG)	[28]
*A. muciniphila*	Obesity	High-fat diet mice	Improved glucose homeostasis, reduced body weight, increased thermogenesis in brown adipose tissue, elevated GLP-1 secretion	[29]
	T2DM	High-fat diet mice	Improved glucose tolerance, reduced weight gain, decreased adipose tissue	[30]
*L. plantarum* CCFM0236	T2DM	High-fat diet, streptozotocin-induced mice	Decreased blood glucose, improved insulin resistance, reduced oxidative stress, and systemic inflammation	[32]
*L. casei*	T2DM	Patients with T2DM	Improved glycemic control, reduced waist circumference, reduced body weight, improved insulin sensitivity	[33]
*B. lactis* V9	NAFLD	High-fat diet mice	Decreased triglycerides, improved glucose metabolism, reduced inflammatory cytokines	[35]
*L. plantarum* NCU116	NAFLD	High-fat diet mice	Restored liver function, reduced oxidative stress, regulated lipid metabolism, decreased fat accumulation in the liver	[37]
